# *Impatiens ngariensis* (Balsaminaceae), a new species from Xizang, China

**DOI:** 10.3897/phytokeys.271.177400

**Published:** 2026-02-10

**Authors:** Ru-Ping Li, Tao-Hua Yuan, Tian Hu, Guang-Wan Hu, Shuai Peng, Qing-Feng Wang

**Affiliations:** 1 College of Ecological Environment, Tibet University, Lhasa 850000, China Wuhan Botanical Garden, Chinese Academy of Sciences Wuhan China; 2 State Key Laboratory of Plant Diversity and Specialty Crops, Wuhan Botanical Garden, Chinese Academy of Sciences, Wuhan 430074, China College of Ecological Environment, Tibet University Lhasa China; 3 Sino-Africa Joint Research Center, Chinese Academy of Sciences, Wuhan 430074, China Sino-Africa Joint Research Center, Chinese Academy of Sciences Wuhan China; 4 Hubei Jiangxia Laboratory, Wuhan 430200, China Hubei Jiangxia Laboratory Wuhan China

**Keywords:** *
Impatiens
*, New Taxon, Phylogeny, Xizang

## Abstract

A new species, *Impatiens ngariensis* (Balsaminaceae) from western Xizang, China, is described in this study. Morphological and phylogenetic evidence supports its taxonomic placement within *I.* sect. *Racemosae*. Phylogenetic analyses indicate that *I. ngariensis* forms a clade with *I. thomsonii*, *I. bomiensis*, *I. fragicolor*, *I. edgeworthii* and *I. glandulifera*. Morphologically, the new species closely resembles *I. sulcata* in petiole with 2 basal glands, subcorymbose-racemose inflorescences, lateral sepals ovate, dorsal petal suborbicular and capsule linear, but can be distinguished by several key characteristics, including a suborbicular dorsal petal with a cordate base and a shorter spur.

## Introduction

The genus *Impatiens* Linnaeus (1753b: 937) is one of the two genera in the family Balsaminaceae, comprising over a thousand species, predominantly distributed across tropical and subtropical mountainous regions of the Old World (Grey-Wilson 1980). Five biodiversity hotspots have been recognised: tropical Africa, Madagascar, southern India-Sri Lanka, Eastern Himalaya and Southeast Asia-southern China (Song et al. 2003; Yuan et al. 2004; Yu et al. 2016; Gogoi et al. 2018). *Impatiens* exhibits extensive floral morphological variation and traditional classification systems, based on macromorphological traits, have largely been established within specific regional contexts. This has led to a slow progression in the development of a comprehensive infrageneric classification. The application of molecular systematics in plant taxonomy has facilitated the integration of molecular and morphological evidence, resulting in a relatively robust and widely applicable infrageneric system (Yu et al. 2016). This system recognises two subgenera, *I.* subg. *Clavicarpa* and *I.* subg. *Impatiens*, with the latter further divided into seven sections.

In China, *Impatiens* currently comprises 358 species (Ding et al. 2022; Hu et al. 2022; Wang et al. 2022; Yuan et al. 2022; Abrahamczyk & Steudel 2023; Huang et al. 2023a, b; Zheng et al. 2023; Tian et al. 2024; Song et al. 2024; Hu et al. 2024; Lyu et al. 2024), predominantly found in south-western China including Yunnan, Sichuan, Xizang and Guizhou (Yuan et al. 2022). As of 2025, more than 70 species of *Impatiens* have been recorded in Xizang and southern Xizang represents one of the major hotspots for the genus in China. All *Impatiens* species recorded from Xizang belong to *Impatiens* subg. *Impatiens* and most of them are assigned to sect. *Racemosae* (Yuan et al. 2022; Tian et al. 2024). In 2024, about eight new species of this genus, including *Impatiens lhunzeensis* J.Tian, G.W.Hu & Q.F.Wang (2024: 52), *I. yingjingensis* X.Q. Song, B.N. Song & Biao Yang (2024: 293), *I. beipanjiangensis* Jian Xu & H.F. Hu (2024: 201), *I. karenensis* Chit Soe Paing & Ruchis. (2024: 113), *I. mogangensis* Y.M. Shui & W.H. Chen (2024: 294), *I. neo-uncinata* Sindhu Arya & V.N.S.A. Kumar (2024: 1), *I. longecauda* Singh & A. Kumar (2024: 87) and *I. minnamparaensis* Sindhu Arya, Ambika, Alen Alex, V.Suresh, Sojan & V.S.A.Kumar. (2024: 83) have been published. These findings underscore the presence of numerous potential new species awaiting discovery within this diverse genus.

During a botanical survey in Xizang in 2023, we discovered an *Impatiens* species along a small stream in Diyag Township, Ngari Prefecture, that closely resembles *I. thomsonii* J. D. Hooker (1859: 128) and *I. sulcata* Wall. (1824: 458). Through detailed morphological comparisons and phylogenetic analyses, we confirmed it as a new species. Additional field investigations and specimen collections were conducted in 2024 and the species is formally described here.

## Materials and methods

### Morphology

Morphological descriptions of new species are based on careful observation of fresh plants and colour photographs, measurements of plants from fresh materials and herbarium specimens. Morphological comparisons were conducted between the new species and morphologically similar species refer to the relevant literature (Wallich 1824; Royle 1835; Marquand 1929; Peng et al. 2020) and colour photographs. Voucher specimens are deposited in HIB. Herbarium abbreviation follows Thiers (2025 ongoing).

### Taxon sampling and DNA sequencing

Phylogenetic reconstruction was performed using two molecular markers (nuclear ribosomal ITS and plastid *atpB*-*rbcL* intergenic spacer) for 146 *Impatiens* species with three outgroup taxa: *Marcgravia umbellata* L. (1753a: 503), *Norantea guianensis* Aublet (1775: 554) and *Hydrocera triflora* (L.) Wight & Arn. (1834: 140). Taxon selection and marker choice followed established methodologies (Yuan et al. 2004; Janssens et al. 2006; Yu et al. 2016). All nucleotide sequences were retrieved from GenBank (complete accession list provided in Suppl. material 1).

Genomic DNA of new species was isolated from silica-gel-dried leaves using the standard CTAB method (Doyle and Doyle 1987). DNA concentration was measured with the Qubit (manufacturer: Invitrogen, model: Qubit 3.0, reagent: Qubit™ dsDNA HS Assay Kit), while integrity was confirmed by 1% agarose gel electrophoresis. Subsequently, a DNA library was constructed using the VAHTS® Universal Plus DNA Library Prep Kit for Illumina (kit model: ND617) and the library was sequenced on the Illumina NovaSeq 6000 sequencing platform with a PE150 sequencing strategy. The chloroplast genome and nrITS sequence was assembled using GetOrganelle v.1.7.5.3 (Jin et al. 2020). To assess the completeness of the final graph, Bandage v.0.9.0 (Wick et al. 2015) was used to visualise assembly graphs.

The phylogenetic relationship was constructed using Maximum Likelihood (ML) and Bayesian Inference (BI) analyses. Phylogenetic analyses were performed in PhyloSuite v.1.2.3 (Zhang et al. 2020). MAFFT v.7.490 (Katoh and Standley 2013) was used to align the sequences with the —auto strategy, which automatically selects the appropriate alignment algorithm and parameters, based on sequence length and similarity. AliView v.1.28 (Larsson 2014) was employed to reverse abnormal sequences, thereby correcting potential directional errors. Subsequently, Trimal v.1.4.rev22 (Capella-Gutiérrez et al. 2009) was utilised to assess the quality of the multiple sequence alignment and accurately excise low-quality regions from the alignment. The two sequences regions, nrITS and *atpB*-*rbcL*, were concatenated. Separate phylogenetic analyses, based on nrITS and *atpB*-*rbcL*, were conducted to assess potential topological incongruence prior to concatenation. The optimal evolutionary models for the nrITS and *atpB*-*rbcL* regions were identified using PartitionFinder v.2.1.1, with both Maximum Likelihood (ML) and Bayesian Inference (BI) methods employed to assess model fit (Guindon et al. 2010; Lanfear et al. 2012; Lanfear et al. 2017). The results indicated that the optimal evolutionary model for the nrITS region was GTR+I+G, whereas the optimal evolutionary model for the *atpB*-*rbcL* region was GTR+G. The ML analysis was conducted using IQ-TREE v.1.6.8. Partitioned analyses were performed, based on a predefined partition scheme, with branch lengths estimated under an edge-linked model. Node support was assessed using 5,000 ultrafast bootstrap replicates, with a minimum correlation coefficient of 0.90 and a maximum of 1,000 iterations. In addition, branch support was further evaluated using the SH-like approximate likelihood ratio test (SH-aLRT) with 1,000 replicates (Minh et al. 2013; Nguyen et al. 2015). The BI analysis was performed using MrBayes v.3.2.7 under a partitioned model framework. Two independent runs were conducted, each consisting of four Markov chains, and run for 10,000,000 generations. Trees and parameters were sampled every 1,000 generations. The first 25% of sampled trees were discarded as burn-in and the remaining trees were used to construct a majority-rule consensus tree to estimate posterior probabilities of clades (Ronquist et al. 2012). Finally, the phylogenetic trees were edited in TreeGraph v.2.15.0–887 beta (Stöver and Müller 2010).

## Taxonomic treatments

### Impatiens ngariensis

Taxon classificationPlantaeEricalesBalsaminaceae

S.Peng, G.W.Hu & Q.F.Wang sp. nov.

CD41C6A0-21F7-5F43-B469-6F83FED76596

urn:lsid:ipni.org:names:77376559-1

**Figure 1.**
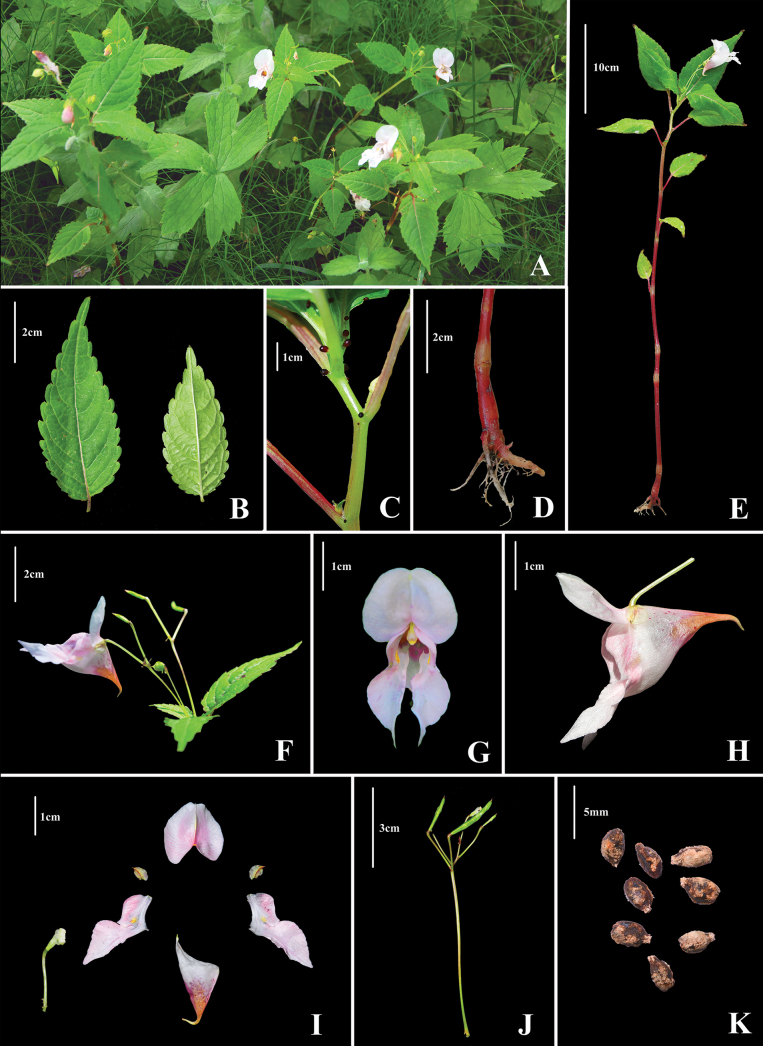
*Impatiens ngariensis*. **A**. Habitat; **B**. Whole plant; **C**. Adaxial and abaxial view of leaf; **D**. Glands; **E**. Roots and basal part of stem, showing node; **F**. Inflorescences; **G**. Front view of flower; **H**. Lateral view of flower; **I**. Dissection of a flower (a. dorsal petal; b. lateral sepals; c. lateral united petals; d. lower sepal; e. stamens and pedicel); **J**. Inflorescences with immature fruit; **K**. Seeds. Photographed by Shuai Peng and Ru-Ping Li.

**Figure 2.**
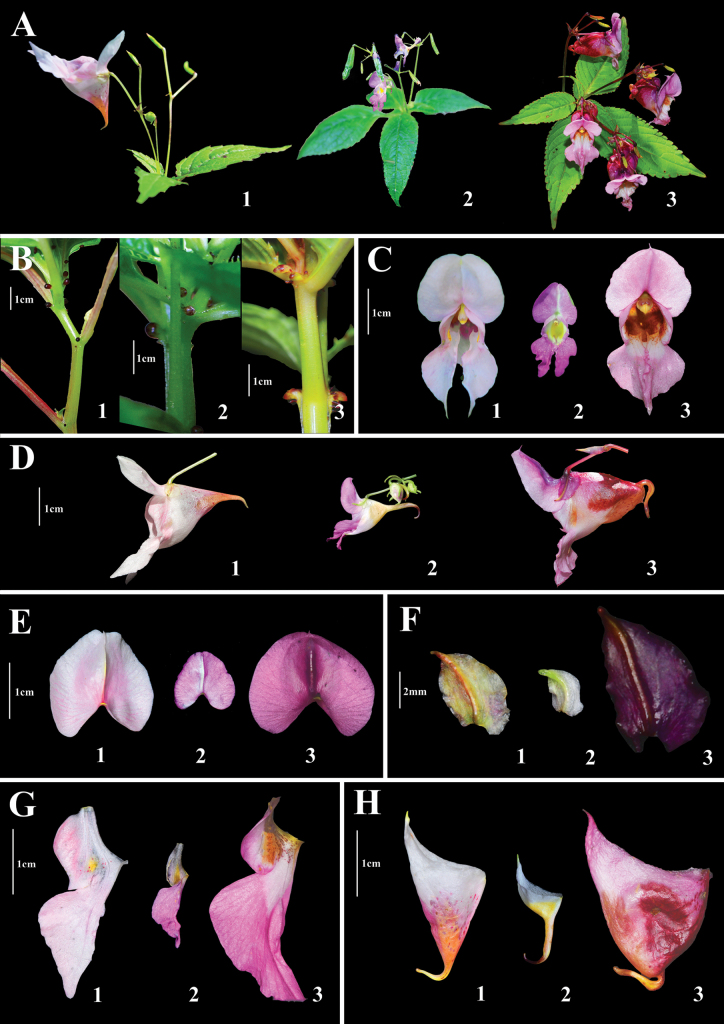
*Impatiens ngariensis* (A1–H1), *I. thomsonii* (A2–H2) and *I. sulcata* (A3–H3). **A**. Inflorescences; **B**. Glands; **C**. Front view of flower; **D**. Lateral view of flowers; **E**. Dorsal petals; **F**. Lateral sepals; **G**. Lateral united petals; **H**. Lower sepals. *Impatiens ngariensis*: photographed by Shuai Peng, *I. thomsonii*: photographed by Shuai Peng (Burang, Ngari Prefecture, Xizang, China) and *I. sulcata*: photographed by Guang-Wang Hu (Yadong, Shigatse, Xizang, China) and Tao-Hua Yuan (Nyalam, Shigatse, Xizang, China).

Fig. 1, Fig. 2: A1–H1

#### Type.

China • Xizang: Ngari Prefecture, Zanda County, Diyag Township, Sibgyi Village, understorey streamside wetlands, 3086 m alt., 31°48'36"N, 78°44'48"E, 15 Aug 2023, *S. Peng, T.H. Yuan, M. Liao & C.Q. Shen PS-00499* (holotype: HIB[HIB0258833]; isotypes: HIB[HIB0258834 and HIB0258835]).

#### Diagnosis.

*Impatiens ngariensis* is morphologically allied to *I. thomsonii* and *I. sulcata*, but differs from the former by its larger flowers, suborbicular (vs. orbicular) dorsal petal and a markedly shorter spur (4–6 mm vs. 1 cm); from the latter, it is distinguished by the oblong (vs. broadly dolabriform to broadly elliptic or ovate) upper petal, dolabriform (vs. subdolabriform to oblong-ovate) lower petal and a broadly funnel-form (vs. saccate) lower sepal.

#### Description.

Annual herbs, 30.5–66.0 cm tall. Stem purplish-red or green, erect, succulent, simple or sparsely branched, glabrous. Leaves alternate; petiole 0.5–2.0 cm, with a pair of deep purplish-red globose or stipitate glands at base; leaf blade elliptic-ovate or oblong-lanceolate, 6.4–12.3 cm × 3.1–3.8 cm, apex acuminate or long acuminate, base cuneate or subrounded, with a pair of stipitate glands, margin crenate-serrate, setose between teeth, lateral veins 6–7 pairs. Inflorescences in upper leaf axils, subcorymbose-racemose, 2–5-flowered; peduncle 2.4–5.5 cm long, pedicel 1.8–2.1 cm long, swollen at apex, bracteate at base; bracts ovate, 0.6–0.7 cm, apex cuspidate, purplish-red or yellowish-green. Flowers white to pale pink, 3.6–4.8 cm long; lateral sepals 2, inequilateral, broadly ovate, 0.6–0.7 cm long, apex apiculate; Lower sepal broadly funnel-form, 1.2–1.6 cm deep, gradually tapering into an incurved, short spur, 0.4–0.6 cm, mouth oblique, 2.8–3.5 cm wide, tip acuminate; dorsal petal suborbicular, ca. 1.9–2.8 cm × 1.8 cm, base cordate, apex emarginate, mucronulate, abaxial mid-vein with a narrow keel; lateral united petals sessile, ca. 2.3–3.0 cm, upper petals oblong, ca. 1.9 cm × 0.9 cm, apex obtuse, lower petals dolabriform, ca. 1.2 cm × 0.8 cm, apex acute, auricle inflexed. Anthers obtuse. Ovary 5-carpellate. Capsules linear, 1.3–2.0 cm, apex rostrum pointed. Seeds ellipsoid, tuberculate.

#### Etymology.

The specific epithet “*ngari*” refers to the type locality, Ngari Prefecture. The Chinese name is given as “阿里凤仙花” (ā-lĬ-fèng-xiān-huā).

#### Phenology.

Flowering late July to August, fruiting August to September.

#### Habitats.

Understorey streamside wetlands at about 3090 m elevation.

#### Distribution.

**Figure 3.**
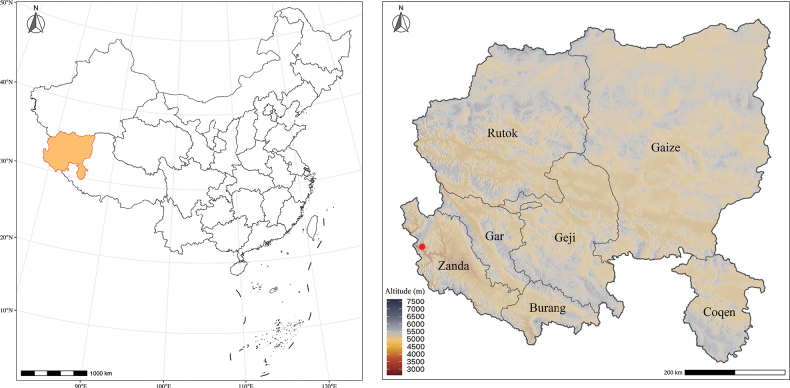
Geographic distribution of *Impatiens ngariensis* in Zanda, Ngari Prefecture (red dots).

*Impatiens ngariensis* is only found in Zanda, Xizang, China (Fig. 3).

#### Conservation status.

The new species is currently known from a single population within China, close to the national border. Due to the lack of comprehensive surveys outside China and insufficient information on its global distribution and population size, it is assessed as Data Deficient (DD), based on International Union for Conservation of Nature (IUCN) Red List Categories and Criteria (IUCN 2025).

#### Phylogenetic position.

**Figure 4.**
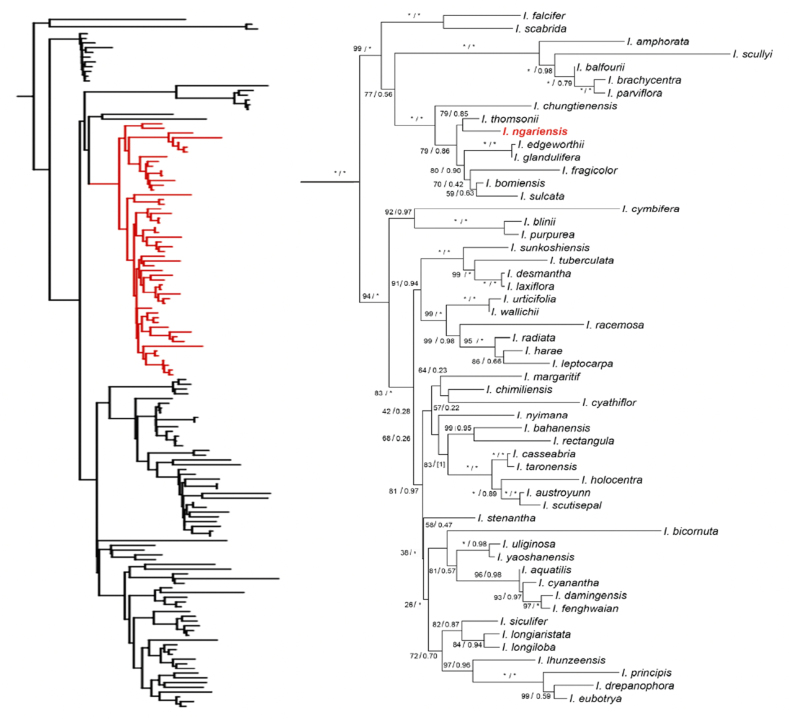
The phylogenetic tree based on a combined dataset of nrITS and plastid *atpB*-*rbcL* DNA sequences. The bootstrap percentages (BP) for Maximum Likelihood (ML) and posterior probabilities (PP) of Bayesian Inference (BI) are shown in the branch (BP/PP, the asterisk [*] indicates BP = 100 or PP = 1.00). Only the ML tree is shown, because its topology is nearly identical to the BI tree. The phylogenetic tree inferred in this study is shown on the left, with the *I.* sect. *Racemosae* clade highlighted in red; the detailed phylogenetic relationships within the *I.* sect. *Racemosae* are shown on the right, and *Impatiens ngariensis* is highlighted in red.

Phylogenetic analysis indicates that the new species is nested within *I.* sect. *Racemosae* and is most closely related to *I. thomsonii*, forming a sister group with it. In addition, they constitute a strongly supported monophyletic clade with six related species: *I. glandulifera*, *I. edgeworthii*, *I. fragicolor*, *I. sulcata*, *I. bomiensis* and *I. chungtienensis*. The clade consisting of the new species and its close relatives is strongly supported by both Maximum Likelihood and Bayesian Inference phylogenetic trees (Fig. 4).

#### Additional specimens examined (paratype).

China. • Xizang: Ngari Prefecture, Zanda County, Diyag Township, Sibgyi Village, understorey streamside wetlands, 3086 m alt., 31°48'36"N, 78°44'48"E, 15 Aug 2023, *S. Peng, T.H. Yuan, M. Liao & C.Q. Shen AL-0459* (HIB); 15 Jul 2024, *J. Tian, T. Hu, J.Z. Su & R.P. Li AL-2383* (HIB).

## Discussion

The morphological characters of *Impatiens ngariensis*, including linear capsules and racemose inflorescences with many flowers and phylogenetic analyses support its placement within *I.* sect. *Racemosae* of *I.* subg. *Impatiens* (Fig. 3). Morphologically, the new species is similar to *I. sulcata* in petiole with two basal glands, subcorymbose-racemose inflorescences, lateral sepals ovate, dorsal petal suborbicular, capsule linear. However, *I. ngariensis* can be easily distinguished from *I. sulcata* by the shape of the lower sepal, the upper petal and the lower petal. *I. ngariensis* can be distinguished from *I. sulcata* by the bracts ovate (vs. lanceolate or ovate-lanceolate), the lower sepal broadly funnel-form (vs. saccate), the upper petals oblong, apex obtuse (vs. subdolabriform to oblong-ovate, apex acute) and the lower petals dolabriform, apex acute (vs. broadly dolabriform to broadly elliptic or ovate, apex acute). Phylogenetically, *I. ngariensis* is closely related to *I. thomsonii*, but *I. ngariensis* can be distinguished from *I. thomsonii* by the inflorescence subcorymbose-racemose (vs. racemose-subcorymbose), the flower large, 3.6–4.8 cm long (vs. small, 0.8–2.1 cm long), the bracts ovate (vs. narrowly lanceolate), the lateral sepals broadly ovate (vs. obliquely ovate), the dorsal petal suborbicular (vs. orbicular), the spur short, 4–6 mm (vs. long, 10–12 mm), the upper petals oblong, apex obtuse (vs. dolabriform, apex rounded), the lower petals dolabriform, apex acute (vs. oblong, apex acute).

Meanwhile, *I. ngariensis* is phylogenetically allied to *I. glandulifera* Royle (1835: 151) and *I. bomiensis* Y.Y. Cong & Y.C. Peng (2020: 2), and can be easily distinguished from these two allied species by the broadly funnel-form lower sepal, the incurved and short spur, the suborbicular dorsal petal, the oblong upper petal and the dolabriform lower petal. The detailed morphological comparisons between new species and four species can be found in Table 1.

**Table 1.** Comparison of *Impatiens ngariensis*, *I. thomsonii*, *I. sulcata*, *I. glandulifera* and *I. bomiensis*.

Characters	*Impatiens ngariensis*	*I. thomsonii*	*I. sulcata*	*I. bomiensis*	*I. glandulifera*
Inflorescence	subcorymbose-racemose, 2–5 flowered	racemose-subcorymbose, few flowered	subcorymbose-racemose, 5–11 flowered	subcorymbose-racemose, 3–7-flowered	racemes, 2–5 flowered
Flower size	3.6–4.8 cm long	0.8–2.1 cm long	3–4.5 cm long	1–2 cm long	2–2.5 cm long
Flower colour	white to pale pink	pinkish	purplish pink	pale pink with purple spots	pink-red or red-purple
Bracts	ovate	narrowly lanceolate, glandular, apex acute	lanceolate or ovate-lanceolate	linear-lanceolate	elliptic-ovate or lanceolate-ovate
Lateral sepals	broadly ovate	obliquely ovate	obliquely ovate-cordate	obliquely ovate	oblique cordate
Lower sepal	broadly funnel-form	broadly funnel-form or saccate	saccate	shallowly funnel-form	saccate
Spur	short spur, 4–6 mm	long spur, 10–12 mm	short spur, 4–8 mm	long spur, 15–19 mm	short spur, 5–6 mm
Dorsal petal	suborbicular	orbicular	suborbicular	suborbicular	orbicular-depressed
Lateral united petals	2.3–3.0 cm, upper petals oblong, apex obtuse, lower petals dolabriform, apex acute	not clawed, upper petals deep pink, dolabriform, apex rounded, lower petals yellow, red spotted, oblong, apex acute	2.8–3.2 cm, upper petals subdolabriform to oblong-ovate, apex acute, cuspidate, lower petals broadly dolabriform to broadly elliptic or ovate, apex acute	1.0–1.5 cm, upper petals subovate, entire, lower petals dolabriform, rostellate at apex	upper petals with a thin incurved appendage, lower petals larger (1.5 cm)

It is noteworthy that a specimen collected from Zanda by the Qinghai-Xizang Expedition Vegetation Group (collection number 12946) and deposited in the Chinese Virtual Herbarium (CVH) was previously misidentified as *I. thomsonii*. Our examination confirms that this specimen represents *I. ngariensis*, highlighting the historical misidentification surrounding this new taxon.

The discovery of this new species not only enriches the diversity of *Impatiens* in this region, but also provides new research materials for exploring the phylogenetic relationships, adaptive radiation and biogeographic patterns of *Impatiens*.

## Supplementary Material

XML Treatment for Impatiens ngariensis

## Additional information

### Use of AI

No use of AI was reported.

### Author ORCIDs

Ru-Ping Li https://orcid.org/0009-0008-8257-7909

Tao-Hua Yuan https://orcid.org/0000-0001-5278-3068

Tian Hu https://orcid.org/0000-0001-6988-9379

Guang-Wan Hu https://orcid.org/0000-0001-7728-7976

Shuai Peng https://orcid.org/0000-0002-8064-920X

Qing-Feng Wang https://orcid.org/0000-0001-9143-8849

### Data availability

All of the data that support the findings of this study are available in the main text or Supplementary Information.

## Supplementary materials

Supplementary material 1 GenBank accession numbers for species in the phylogenetic analysis Data type xlsx https://binary.pensoft.net/file/1536644This dataset is made available under the Open Database License (http://opendatacommons.org/licenses/odbl/1.0/). The Open Database License (ODbL) is a license agreement intended to allow users to freely share, modify, and use this Dataset while maintaining this same freedom for others, provided that the original source and author(s) are credited. Ru-Ping Li, Tao-Hua Yuan, Tian Hu, Guang-Wan Hu, Shuai Peng, Qing-Feng Wang
